# GFRα 1-2-3-4 co-receptors for RET Are co-expressed in Pituitary Stem Cells but Individually Retained in Some Adenopituitary Cells

**DOI:** 10.3389/fendo.2020.00631

**Published:** 2020-09-24

**Authors:** Alberto Pradilla Dieste, Miguel Chenlo, Sihara Perez-Romero, Ángela R. Garcia-Rendueles, Maria Suarez-Fariña, Montserrat Garcia-Lavandeira, Ignacio Bernabeu, José Manuel Cameselle-Teijeiro, Clara V. Alvarez

**Affiliations:** ^1^Neoplasia & Endocrine Differentiation P0L5, Centro de Investigación en Medicina Molecular y Enfermedades Crónicas (CIMUS), Instituto de Investigación Sanitaria (IDIS), University of Santiago de Compostela (USC), Santiago de Compostela, Spain; ^2^Department of Endocrinology, Complejo Hospitalario Universitario de Santiago de Compostela (CHUS)-SERGAS, Instituto de Investigación Sanitaria de Santiago (IDIS), USC, Santiago de Compostela, Spain; ^3^Department of Pathology, Complejo Hospitalario Universitario de Santiago de Compostela (CHUS)-SERGAS, Instituto de Investigación Sanitaria de Santiago (IDIS), Santiago de Compostela, Spain

**Keywords:** pituitary stem cell niche, GPS, RET, progenitors, cell turnover

## Abstract

The RET tyrosine kinase receptor is expressed by the endocrine somatotroph cells of the pituitary where it has important functions regulating survival/apoptosis. However, RET is also expressed by the GPS pituitary stem cells localized in a niche between the adenopituitary and the intermediate lobe. To bind any of its four ligands, RET needs one of four co-receptors called GFRα1-4. It has been previously shown that GFRα1 is expressed by somatotroph cells and acromegaly tumors. GFRα2 was shown to be expressed by pituitary stem cells. GFRα4 was proposed as not expressed in the pituitary. Here we study the RNA and protein expression of the four GFRα co-receptors for RET in rat and human pituitary. The four co-receptors were abundantly expressed at the RNA level both in rat and human pituitary, although GFRα4 was the less abundant. Multiple immunofluorescence for each co-receptor and β-catenin, a marker of stem cell niche was performed. The four GFRα co-receptors were co-expressed by the GPS cells at the niche colocalizing with β-catenin. Isolated individual scattered cells positive for one or other receptor could be found through the adenopituitary with low β-catenin expression. Some of them co-express GFRα1 and PIT1. Immunohistochemistry in normal human pituitary confirmed the data. Our data suggest that the redundancy of GFRα co-expression is a self-supportive mechanism which ensures niche maintenance and proper differentiation.

## Introduction

RET (HGCN approved name: ret proto-oncogene) is a tyrosine kinase receptor involved in cell survival, proliferation, and differentiation. RET is single pass transmembrane protein essential for tubular kidney and digestive parasympathetic nervous system embryonic development (1). On the other hand, RET signaling pathways have a crucial role in the postnatal pituitary plastic capacity (1). For ligand binding and subsequent activation, RET needs a co-receptor from the GFRα family (GDNF family receptor alpha). There are four RET ligands with special affinity for one specific co-receptor. GFRα1 has affinity for Glial cell-derived neurotrophic factor (GDNF), GFRα2 for neurturin (NTN), GFRα3 binds artemin (ART), and GFRα4 needs persephin (PSP) [see STRING, https://version-11-0.string-db.org/cgi/network.pl?networkId=lhJBsGLDouUC; reviewed in (1)]. Despite the special affinity of each co-receptor with a specific RET ligand, there is some promiscuity between them. GFRα1 and GFRα2 could be cross-activated by NTN and GDNF, respectively, due to similarities in structure (2–5). GFRα2 can also activate p140NCAM (Neural cell adhesion molecule 1) in neurons lacking RET (6). GFRα4 also responds to NTN in high concentrations (7). On the other hand, GFRα3 seems just activated by ART (3). A fifth RET co-receptor, GFRAL (GDNF Family Receptor Alpha Like), has been described having GDF15 (Growth differentiation factor 15) as ligand but there is no GFRAL interaction with other RET ligands, nor GDF15 binding to GFRα1–4 co-receptors (8–11). The pituitary gland is a neuroendocrine organ performing as the main governor of the endocrine system. The gland is composed by three parts: anterior lobe (adenopituitary, AP), intermediate lobe (IL) and posterior lobe (neuropituitary, NP). The five hormone-secreting endocrine cells are localized in the AP, somatotrophs (growth hormone, GH), thyrotrophs, (thyroid stimulating hormone, TSH), lactotrophs (prolactin, PRL), corticotrophs (adrenocorticotropin, ACTH), and gonadotrophs (follicle stimulating hormone, FSH; and luteinizing hormone, LH). Each of these cell lineages express a characteristic transcription factor that induce and maintain cell differentiation (12–14). The adult pituitary gland contains an adult stem cell niche formed by two rows of cells located in the marginal zone (MZ), between the AP and the IL (15, 16).

Within the adenopituitary, somatotrophs express both RET and GFRα1, together with GDNF and this is maintained in somatotroph-derived pituitary tumors causing acromegaly (17–19). RET is implicated in two pathways that control the number of somatotroph cells: in the absence of ligand GDNF, the RET/PIT1/CDKN2A-p14ARF/p53 pathway leads to apoptosis while in the presence of ligand the RET/GDNF/AKT leads to survival (1, 19–22). Thus, RET can act as a dependence receptor (absence of ligand) or tyrosine-kinase (presence of ligand) (23). RET could be also expressed in female lactotrophs during the different pregnancy/lactation stages, in a similar pathway regulating lactotroph apoptosis after weaning (24).

Interestingly, postnatal pituitary stem cells also express RET together with high levels of the co-receptor GFRα2, and with the ontogeny transcription factor PROP1 (HGCN approved name: PROP paired-like homeobox 1; previously called Prophet of Pit1), and many stem-cell markers, defining its name as the GPS cells (GFRα2, PROP1, Stem) (15). As other stem cells, pituitary stem cells express stemness related markers like SOX2 (SRY-box transcription factor 2), SOX9 (SRY-box transcription factor 9), KLF4 (Kruppel like factor 4), and POU5F1 or OCT4 (POU class 5 homeobox 1) (15, 25, 26). GPS are organized in a pituitary stem-cell niche associated to supportive cells from where they are recruited for transit-amplification, commitment, and differentiation (13, 15, 27–29).

On the other hand, GFRα4 was originally described as not expressed in human pituitary (30). However, *GFRA4* mRNA has recently been shown to be expressed in somatotroph pituitary tumors causing acromegaly (19). *GFRA4* was significantly correlated with poorer prognosis and resistance to first-line therapy. These somatotroph tumors also expressed some *PROP1* mRNA, a stem-cell transcription factor that is not detected in normal somatotroph cells.

The apparent contradictions related to *GFRA4* expression in the altered somatotroph adenomas while it seems not expressed in the normal pituitary, the possibility that GFRα co-receptors can function independently of RET together with the possibility that co-expression of the RET co-receptors could be essential to define stemness in the pituitary drove us to make a comparative study of the four GFRα receptors in the pituitary. RNA and protein expression of each co-receptor was assessed in human and rat pituitary, aiming to describe their distribution among the lobes of the pituitary gland.

## Materials and Methods

### Pituitary Samples

Male and female young adult *Sprague-Dawley* (90 days old) rats were purchased in the registered facility of our institution (CEBEGA). Male and female 90 days old *Wistar Han* rats were purchased from Janvier Labs. Rats were perfused and the pituitary was immediately dissected and post-fixed overnight in 4% paraformaldehyde. Procedures were carried out under license to CVA granted by the corresponding legal authority in animal research within the Galicia Regional Government.

The human pituitary sample was obtained after informed consent from the Biobank of Complejo Hospitalario Universitario de Santiago de Compostela (CHUS). It was a 55 y.o. male patient dead from colon cancer immediately upon admission and did not received any previous therapy.

### RNA Extraction

The rat pituitary was dissected after perfusion, discarding the neuropituitary. Adenopituitary (AP) together with Intermediate Lobe (IL) were frozen at −80°C. RNA extraction was performed using the TRIzol™ reagent (15596026, Invitrogen), following manufacturer instructions. A commercial human Pituitary Gland Poly A+ mRNA pool (1305204A, Clontech, USA) was used. The pool comes from 88 normal pituitary glands of Caucasian men and women aged 16–68 years who died from sudden death.

### qRT-PCR Assay

One microgram of total RNA were incubated with 1 IU RNase free DNase I (EN0521, Thermo), 5 μL 10X buffer with MgCl_2_ and water for a total volume of 50 μL, at 37°C for 30 min. The reaction was terminated by inactivating DNase and then RNA was purified with an affinity column using the GeneJET RNA Cleanup and Concentration micro kit (K0842, Thermo Fisher). RNA was finally quantified by spectrophotometry (Nanodrop 2000, Thermo Fisher). Previous to cDNA synthesis, we performed a pre-treatment with DNase incubating 1 μg of RNA with 1 IU of RNase-free DNase I (EN0521, Thermo Fisher), 1 μL of MgCl_2_ buffer and water to a final volume of 10 μL for 30 min at 37°C. DNAse was then inactivated by adding 1 μL of EDTA and incubating for 10 min at 65°C. cDNA was synthesized following the supplier's protocol, adding 1.5 μL of 300 IU MMLV (28025-013, Invitrogen, USA), 6 μL 5X First-Strand Buffer, 1.5 μL 10 mM dNTPs, 0.1 μL Random Primers, 3 μL 0.1 M DTT, 1 μL RNaseOUT™ Recombinant Ribonuclease Inhibitor (40 units/μL) and H_2_O for a total 30 μL reaction. For human samples, 50, 25, and 12.5 ng of Poly A+ mRNA was similarly treated.

Expression was detected by qPCR using 1 μL of the cDNA reaction plus 6 μL 2x TaqMan Gene Expression MasterMix (4369016 Applied Biosystems) and 6 μL diluted primers in 96 well-plates in a 7500 Real-Time PCR System (4351105, Applied Biosystems, USA). Primers and TaqMan assays used for each gene were designed in contiguous exons and are summarized in Supplementary Table 1. As control for general gene expression we used human or rat *TBP* based in published works (19, 31, 32). Negative controls of the reverse-transcription step (all reagents and RNA sample but without reverse transcriptase) and the PCR step (all reagents but no reverse-transcribed sample) were included in each assay plate.

### Rat Pituitary Immunofluorescence

For rat pituitary we used coronal sectioning of formalin-fixed paraffin-embedded male pituitaries in 4-micron sections. For dewaxing sections were incubated overnight at 52°C. Antigen retrieval was done using the PT-Link system in high pH buffer (DAKO-Agilent). Sections were incubated with primary antibodies overnight at 4°C and then 1 h with secondary antibodies at room temperature. For nuclei staining we used DAPI. Concentrations and conditions of antibody incubations are listed in Supplementary Table 2. Slides were photographed using a confocal microscope TC-SP5-AOBS with a white laser (470–670 nm) and a UV laser (Leica) with LAS AF (*Leica Application Suite Advanced Flourescence*) software, using serial sections (Z) every 1 μm for the 20X (PL APO 20X/N.A.0.70 CS) objective and 0.3 μm for the 63X objective (oil PL APO 63x/N.A.1.4-0.6 CS). Data were collected using Sequential Mode with the following order: first, Channel 00 (DAPI); second, Channel 02 (Alexa 488); third, Channel 03 (Cy3). Data were collected at resolution 1,024 × 1,024 pixels, with zoom 1x-3x, giving an XY field of a range from 775 × 775 μm till 288.5 × 288.5 μm for objective 20x and of a range of 246 × 246 μm till 90.4 × 90.4 μm for objective 63x. Thus, the final resolution was between 0.75 and 0.28 μm/pixel (20x) and 0.24–0.08 μm/pixel (63x).

### Immunohistochemistry

We used 3 μm sagittal sectioning of the formalin-fixed paraffin-embedded pituitary. Sections were deparaffinised and retrieved as above. Sections were blocked with EnVision® FLEX Peroxidase-Blocking Reagent (DAKO-Agilent). Staining was performed incubating with primary antibody in diluent buffer followed by incubation with EnVision™ FLEX/HRP system (DAKO-Agilent) followed by DAB. Nuclei were counterstained with diluted Hematoxylin. Concentrations and conditions of antibody incubations are listed in Supplementary Table 2.

### Graphs and Descriptive Statistics

Results are presented as Mean ± SEM using GraphPad Prism v.7 (GraphPad Software, California USA). Normality for each group of quantitative data was assessed using the Kolmogorov-Smirnov test with Dallal-Wilkinson-Lillie for “*p*” value. Quantitative variables were compared by Mann-Whitney tests when non-parametric or unpaired *t*-tests when parametric.

## Results

### The Four GFR Alpha co-receptors of RET Are Expressed in Rodent and Human Pituitary

**Figure 1:**
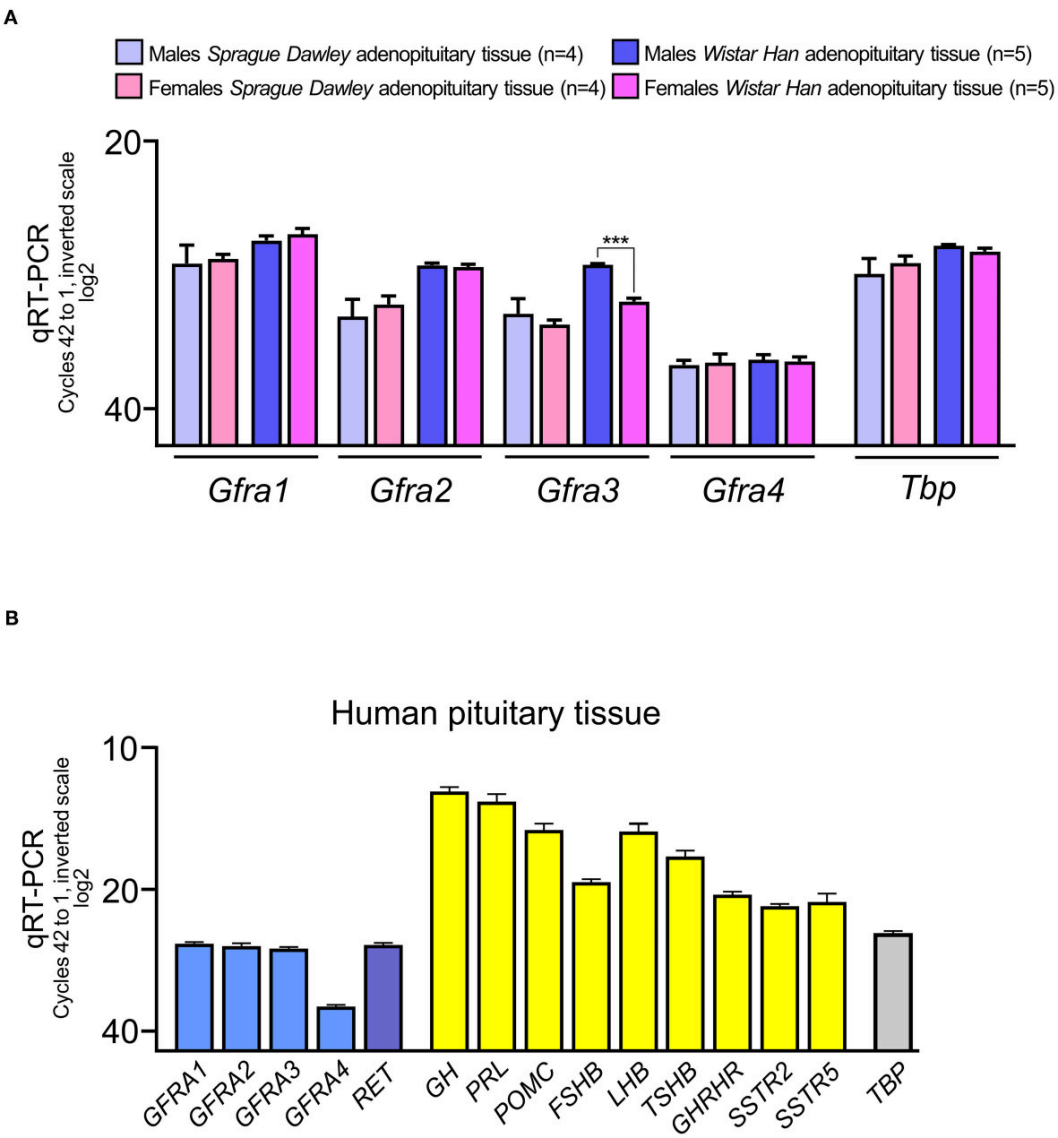
RNA expression of the *GFRA* co-receptors of RET in the adenopituitary. For comparative purposes among different genes, expression is shown in cycles of the qRT-PCR compared to the housekeeping control gene, *Tbp* (in rat pituitary) or *TBP* (in human pituitary). The total number of cycles is 40. Less cycles required for amplification, closer to 20 cycles, higher expression since the scale is log2. **(A)** The four co-receptors are expressed in male and female adenopituitary from the two most common strains used in biomedical research, Sprague-Dawley, and Wistar. The most abundant co-receptor is *Gfra1* and the least expressed is *Gfra4*. Female Wistar adenopituitary express significantly less *Gfra3* than males. **(B)** Expression of *GFRA* co-receptors and *RET* in human normal pituitary, pooled from men, and women. For comparative purposes hormones and receptors for hypothalamic factors were also measured. As in rat pituitary, *GFRA4* is the less abundant in human adenopituitary.

To clarify which of the four co-receptors were expressed in the pituitary we performed quantitative RT-PCR for each co-receptor. In rats, we used AP of young adult male and female rats (90 days old) of the two most common strains, *Sprague-Dawley* and *Wistar*. As expected from our previous results in Sprague-Dawley rats (15, 17), expression of the co-receptor *Gfra1* and *Gfra2* was abundant in rat AP without differences between male and female (Figure 1A). No differences were found when compared to *Wistar* rats. We could detect expression of the two other co-receptors *Gfra3* and *Gfra4*, this last being the least expressed in both rat strains. We did not find sex differences in expression except for *Gfra3* significantly less expressed in *Wistar* females (Figure 1A).

Next we studied the expression of the four RET co-receptors in the human pituitary (Figure 1B). A mixed pool of male and female adult normal adenopituitaries was used. To establish the quality of the pool, we detected the expression of hormones and receptors for some hypothalamic factors. As expected the most abundant detection corresponded to GH and PRL, since the normal pituitary has a ratio of more than 50% of somatotrophs and around 35% of lactotrophs, with certain variations in women (proportionally more lactotrophs) respect to men (13, 15, 18, 21, 33). The next hormone expressed was POMC corresponding to corticotrophs being the next in number (around 15%). Gonadotrophs are around 10% of endocrine cells and express both *LHB* and *FSHB*; *LHB* expression is as abundant as *POMC* but *FSHB* is less expressed. Thyrotrophs are the least abundant endocrine population but *TSHB* is as well-expressed as *FSHB*. This indicates that in addition to the proportion of cells of each type, other factors must be considered, such as the direct regulation of the expression of that gene. Both *GHRH, SSTR2*, and *SSTR5* were abundant.

*RET* and the four *GFRA* co-receptors are expressed in the human AP at levels slightly under the housekeeping gene *TBP*. Interestingly, the co-receptor GFRA4 that previously had been suggested to be absent, is clearly expressed although as happened in the rat AP, it is the least abundant GFRα co-receptor.

### Functional Localization of RET and Its Four co-receptors in Rat Pituitary

Once we were sure that all four RET co-receptors were expressed at the mRNA level, we studied its protein expression within the pituitary gland. For the rat pituitary, we used immunofluorescence and confocal microscopy. Since we did not find differences in mRNA expression, we used male pituitaries. The major cell population in the pituitary are the endocrine cells grouped in acini within the AP. A second population, the pituitary stem cells are localized at a niche comprising the first cell layer between the IL and AP (13, 15, 16, 25). In this niche, the parenchymal cells are the GPS cells called, as described above, for the acronym of some co-expressed proteins: GFRα2, PROP1 and Stem Cell factors (SOX2, SOX9, and OCT4). The niche also contains other cell types supporting the GPS (15). The niche projects into the AP as finger like projections appearing as rounded structures in a section (13, 34).

**Figure 2:**
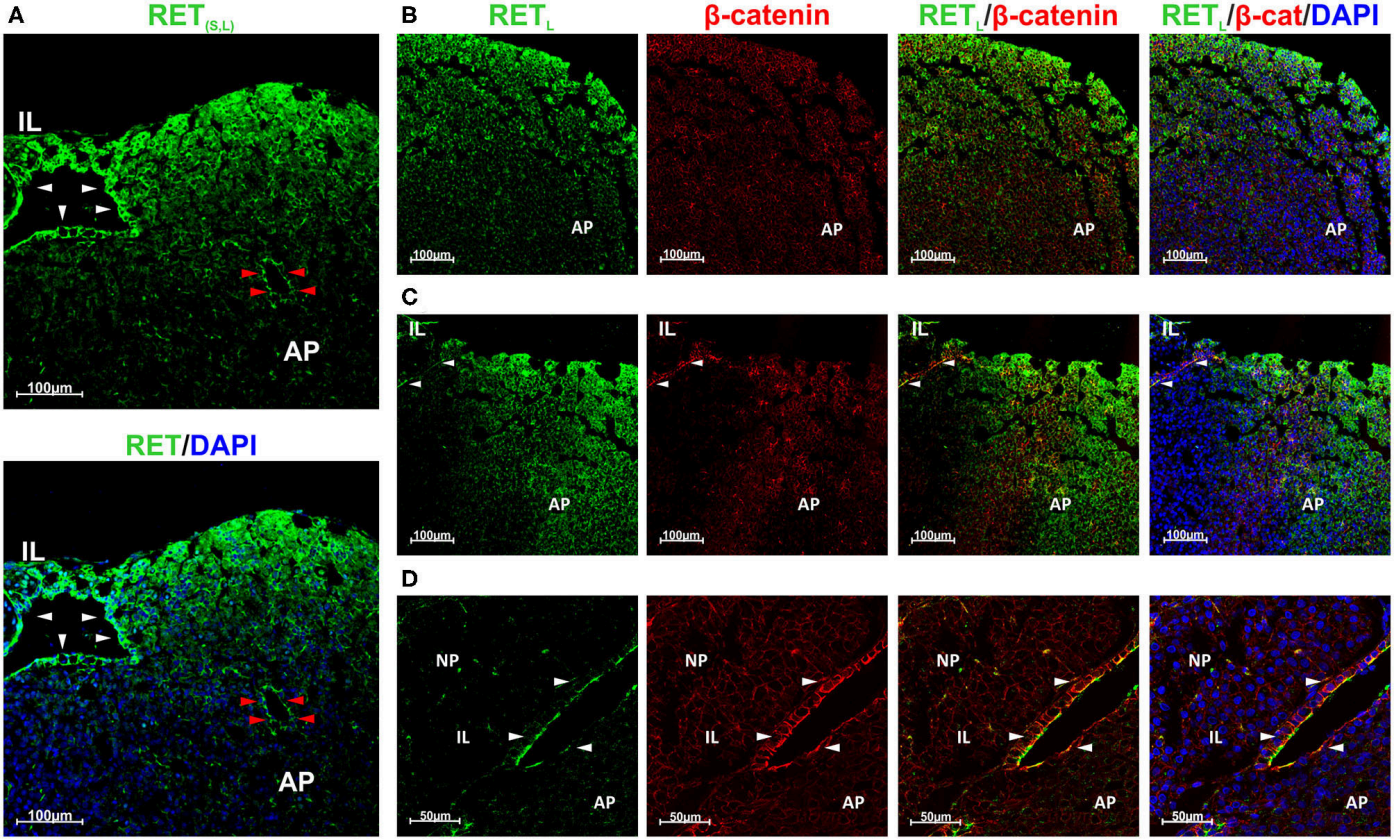
GPS cells at the pituitary stem cell niche co-express RET and β-catenin. RET is also expressed by endocrine cells at the periphery of the AP lobes. **(A)** High RET expression (green) in the stem cell niche (white arrowheads) and in one follicle within the AP (red arrowheads). It was detected with an antibody recognizing both isoforms (S and L). RET is also expressed at the periphery of the AP lobe. **(B–D)**: RET was detected with an antibody for RET_L_ isoform. **(B)** At the AP, RET (green) is expressed at the periphery of the lobe and do not colocalize with β-catenin (β-cat, red). DAPI (blue) is used to reveal the nuclei. **(C)** At the marginal zone between the IL and the AP the GPS cells reside, forming the pituitary niche. RET (green) and β-catenin (red) are co-expressed in GPS showing yellow blended color (arrowheads). In the AP, RET does not colocalize with β-catenin. **(D)** Higher magnification at the niche, shows colocalization of RET (green) and β-catenin (red) at the GPS cells (yellow at the combined projection, indicated by arrowheads). AP, Adenopituitary; IL, Intermediate Lobe; NP, Neuropituitary.

We have previously studied RET expression in the endocrine cells of the AP restricting it to the somatotrophs (17). However, we had not compared the expression between the niche and the somatotrophs. Shown in Figure 2 is a coronal rat pituitary section. RET is expressed at the outside cell layers of the AP lobe where somatotrophs are located but it is expressed more intensely in the stem cell niche (Figure 2A). Moreover, the follicles within the AP also present higher RET expression. Here it was detected with an antibody recognizing the two RET_L_ and RET_S_ isoforms. There are two RET isoforms, RET_L_ and RET_S_ with a small difference in protein length at the end of the cytoplasmic C-terminal tail. Both are equally expressed in the pituitary (15, 19, 20). To further associate this high intensity of expression with the stem cell niche we co-stained for RET_L_ and β-catenin. It is known that GPS cells of the pituitary niche express huge levels of β-catenin a characteristic shared with embryonic stem cells (33, 35–37). On the other hand, β-catenin is expressed at low levels by all epithelial cells including the endocrine AP cells (Figures 2B,C) (38, 39). At the niche, RET –detected with RET_L_ antibody- was co-localized with β-catenin both expressed at high levels (Figure 2C). Intriguingly, RET expression was concentrated at the apical pole of the GPS cell layer (Figure 2D).

**Figure 3:**
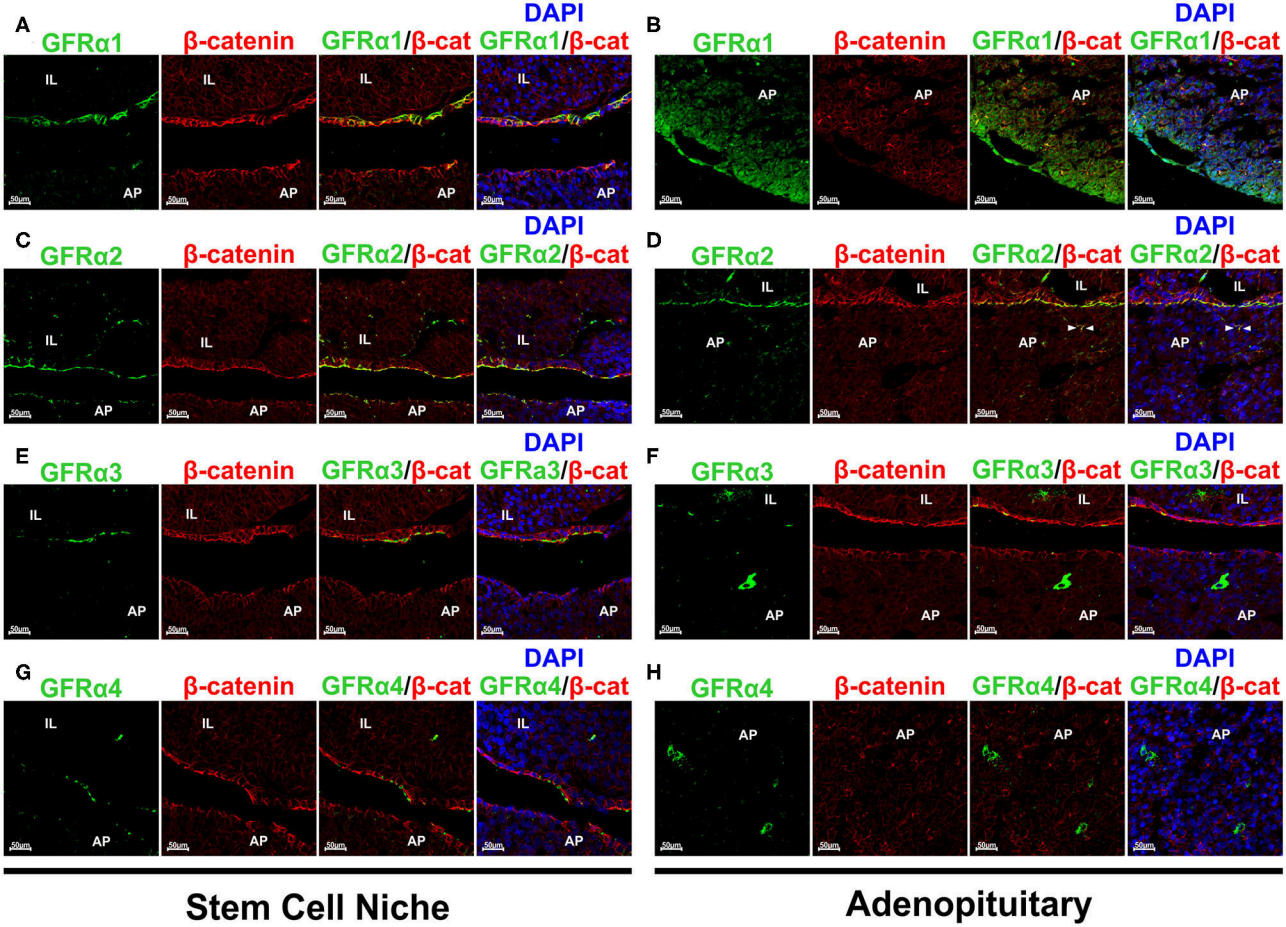
Expression of the GFRα 1-2-3 and 4 co-receptors in rat pituitary. Sections were double stained for each one of the co-receptors and β-catenin to reveal the pituitary stem cell niche. **(A)** GFRα1 (green) colocalizes with β-catenin (β-cat, red) at the GPS niche (yellow blended color in the combined projection). However, GFRα1 cells at the adenopituitary do not co-express β-catenin **(B)**. DAPI is shown to reveal the nuclei. **(C)** GFRα2 is continuously expressed along the pituitary stem cell niche, at the apical pole of the GPS cells. It is co-expressed with β-catenin. **(D)** There are few GFRα2 cells through the AP and are also GPS cells located at the pituitary follicles as shown by co-expression with β-catenin (arrowheads). **(E)** GFRα3 is also co-expressed with β-catenin at the apical pole of the GPS cells. **(F)** There are isolated GFRα3 expressing cells that do not express β-catenin. **(G)** GFRα4 is also colocalized with β-catenin at the niche. **(H)** Isolated GFRα4 cells negative for β-catenin were found in the AP (Left) but also—and numerous- in the IL (Right). AP, Adenopituitary; IL, Intermediate Lobe; NP, Neuropituitary.

Our next step was to establish the staining for each of the GFRα co-receptors, looking at whether there was staining at the GPS niche or at the adenopituitary or both. GFRα1+ was expressed in the stem cell niche as demonstrated by its colocalization with β-catenin (Figure 3A). In the adenopituitary, cells expressing GFRα1 negative for β-catenin were localized at the AP lobes outside the niche (Figure 3B).

The GFRα2 co-receptor shows a continuous staining of the apical pole of the first cell layer in the marginal zone of in the cleft surrounding IL and AP (Figure 3C). It is fully colocalized with β-catenin (Figure 3C). GFRα2 is also expressed in a layer of cells surrounding groups of IL cells, at what we consider an extension of the pituitary niche (Figure 3C). GFRα2 is considered one of the principal markers for pituitary stem cells, those known as GPS (15). Only a small population of weak GFRα2 positive cells are scattered within the adenopituitary (Figure 3D). This expression is related to partial sectioning through pituitary follicles since they show blended signal with β-catenin (Figure 3D).

GFRα3 was also expressed at the apical pole by the niche cells co-expressing beta-catenin (Figure 3E), as previously published (39). However, contrary to GFRα2, GFRα3 expression seems less abundant in comparison to GFRα2 since its expression was not continuous. Isolated GFRα3 positive cells, with intense cytoplasmic staining and negative for β-catenin were dispersed throughout the AP (Figure 3F). These cells did not express β-catenin suggesting that they were differentiated cells or cells in the process of differentiation.

The last of the RET co-receptors was GFRα4 that presented a pattern similar to the one found for the other co-receptors at the niche (Figure 3G). GFRα4 was in the extracellular aspect of the plasma membrane in GPS cells co-expressing β-catenin. Regarding the AP, GFRα4 was expressed in isolated cells negative for β-catenin showing a cytoplasmic staining (Figure 3H, left). Moreover, abundant cells expressing GFRα4 were found at the IL (Figure 3H, right).

**Figure 4:**
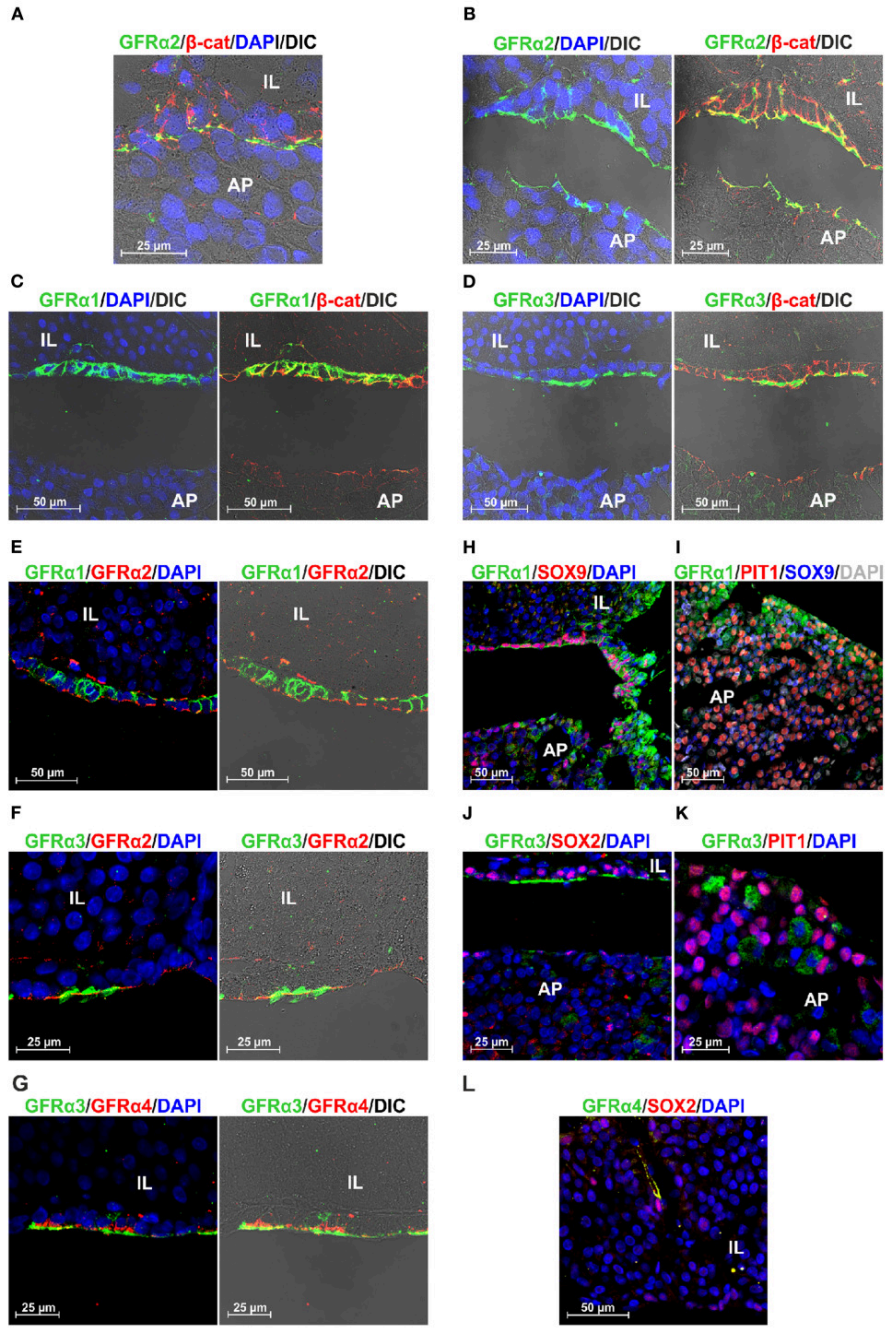
Polarization and colocalization of the 4 GFRα at the niche but not in the AP. GFRα1 is also expressed in PIT1+ cells. **(A,B)** Colocalization of GFRα2 and β-catenin (β-cat) at higher magnification in a rat pituitary sample that maintain its structure with a thin MZ **(A)**, compared to another that presents the usual fixation-induced retraction opening the MZ **(B)**. **(B)** GFRα2 accumulates in the apical pole of the GPS cells residing in the stem cell niche, oriented toward the AP. **(C)** In contrast to GFRα2, GFRα1 is distributed along the plasma membrane of GPS cells. **(D)** GFRα3 coincides with GFRα2 in the apical pole oriented toward the AP. **(E)** Colocalization of GFRα1 and GFRα2 in the GPS cells of the niche. **(F)** Colocalization of GFRα3 and GFRα2 in the GPS cells of the niche. GFRα3 appears outside of GFRα2 in what resembles cilia in the DIC. **(G)** Colocalization of GFRα3 and GFRα4 in the GPS cells of the niche. GFRα4 is also polarized toward the AP. GFRα3 is outside of GFRα4. **(H)** In the niche, GPS cells contain nuclear SOX9 and GFRα1 highly enriched in the plasma membrane. As we enter the AP, a border area known as the wedge, GFRα1 staining spreads throughout the cell, which is no longer SOX9 positive. **(I)** The periphery of the AP lobes is a characteristic zone of somatotroph cells. There we found abundant GFRα1+ (green) /nuclear PIT1+ (red) cells but very few GFRα1+/nuclear SOX9+ (blue). Some cells show nuclear PIT1 and cytoplasmic SOX9, which is sometimes combined with GFRα1. Some cells are double negatives for PIT1/SOX9 (gray nucleus). **(J)** GFRα3+ cells colocalize with nuclear SOX2 in the niche, but are negative for SOX2 in the AP. **(K)** GFRα3+ cells do not co-express PIT1. **(L)** In IL, an elongated GFRα4+ cell with cytoplasmic SOX2 just next to a cell with intense nuclear SOX2.

To further confirm these results we performed new stainings and recorded it at higher magnifications, adding an overlay with transillumination using Differential Interference Contrast (DIC) that reveals tissue structure. The space between the niche containing the GPS cells and the AP, the MZ, is thin and present some actual contacts between both layers. However, fixation and embedding retracts the tissue separating both layers, MZ and AP, allowing a more detailed study of the GPS cells. We know this by our previous studies in GFRα2, the most well-studied co-receptor (13, 15). Figure 4A shows GFRα2 and β-catenin in an unusual pituitary that remained with a thin MZ. Figure 4B shows the usual open MZ where the niche remains stuck to the IL. GFRα2 is localized at extracellular aspect of the plasma membrane in the apical pole of the GPS cells, with some cells (or fragments) remaining at the AP side after the retraction, indicating its proximity in the pituitary *in vivo*. Another layer of GFRα2 is present at the apical pole of the IL (see below). In contrast to GFRα2, GFRα1 is distributed all around the plasma membrane of the GPS cell delimitating its shape like β-catenin (Figure 4C). GFRα3 (Figure 4D) and GFRα4 (see below) are less expressed but coincide in the apical pole with GFRα2. Thus, RET, GFRα2, GFRα3, and GFRα4 are all of them apical proteins.

The localization of these proteins suggested that all co-receptors were co-expressed together with RET in GPS niche cells. We carried out new stains aimed specifically at studying this to the extent of technical possibilities: two of the antibodies had rabbit origin (GFRα2 and GFRα4) and the other two goat origin (GFRα1 and GFRα3), precluding double stainings among themselves. We observed colocalization of GFRα1 and GFRα2 the two most expressed receptors all along the GPS cell layer (Figure 4E). GFRα3 is colocalized with GFRα2 at the apical pole, and it is concentrated in structures suggesting cilia (Figure 4F). GFRα4, the less expressed receptor, is colocalized with GFRα3 at the apical pole but, with an appearance similar to GFRα2, in the plasma membrane behind GFRα3 (Figure 4G).

We could not find any colocalization of these receptors outside the niche in the adenopituitary although, as shown above in Figure 3, there were cells expressing GFRα co-receptors. To explore the possibility that these cells were differentiating or already differentiated we stained with some nuclear markers. SOX2 and SOX9 are transcription factors highly co-expressed by the GPS cells at the niche, but remaining in cells that leave the niche committed to differentiation (27, 28). PIT1 is the transcription factor characteristic of differentiated somatotrophs, lactotrophs, and thyrotrophs. The most abundant co-receptor within the AP is GFRα1 that is expressed together with RET and GDNF in many somatotroph cells (18). Figure 4H shows the transition between the MZ and the AP, so-called the wedge. At the niche, SOX9+ GPS cells express GFRα1 at the plasma membrane; as the AP is reached, GFRα1 cells show more diffuse staining throughout the cytoplasm, and most are negative for SOX9. Somatotroph cells are concentrated on the periphery of the AP lobes. In this zone, most of the GFRα1 cells are PIT1+ (Figure 4I); few GFRα1 cells have a SOX9+ nucleus. Interestingly, many PIT1+ cells have cytoplasmic SOX9, and in some this is added to cytoplasmic GFRα1 (Figure 4I).

GFRα3 colocalized with SOX2 at the niche but not in the AP (Figure 4J). GFRα3 also did not colocalized with PIT1 (Figure 4K). GFRα4 did not colocalize with PIT1 or SOX9 in the pituitary gland. We found at the IL some elongated cells co-expressing GFRα4 and cytoplasmic SOX2 in the vicinity of another intense SOX2+ (Figure 4L).

### Functional Localization of RET and Its Four co-receptors in Human Pituitary

The results obtained in the rat pituitary indicated that the GPS cells of the marginal zone co-expressed all co-receptors GFRα 1-2-3 and 4 together with RET plus the stemness markers (SOX2, SOX9, PROP1, OCT4). On the other hand, within the AP, isolated cells outside the niche expressed only one of those co-receptors, either while differentiating or during transiently amplifying after recruitment. Previous studies have shown that the human pituitary expresses RET together with GFRα1 in the somatotrophs (18) and the co-receptor GFRα2 together with OCT4 at the stem cell niche (15). As indicated above, human somatotroph adenomas have expression of the GFRα4 co-receptor (19). This led us to also study the expression of the four RET co-receptors in the normal human pituitary.

It is not easy to obtain human pituitary glands from healthy and middle-aged adults in hospital tissue banks. The pituitary must be preserved whole and well-oriented to study the stem cell niche, the patient must not be elderly because the niche may no longer be functional, and the cause of death or previous treatments should not affect the pituitary or to the conservation of the sample. Our case was a 55-year-old male patient whose death was upon admission to the hospital due to causes other than pituitary pathology, and no previous treatments.

**Figure 5:**
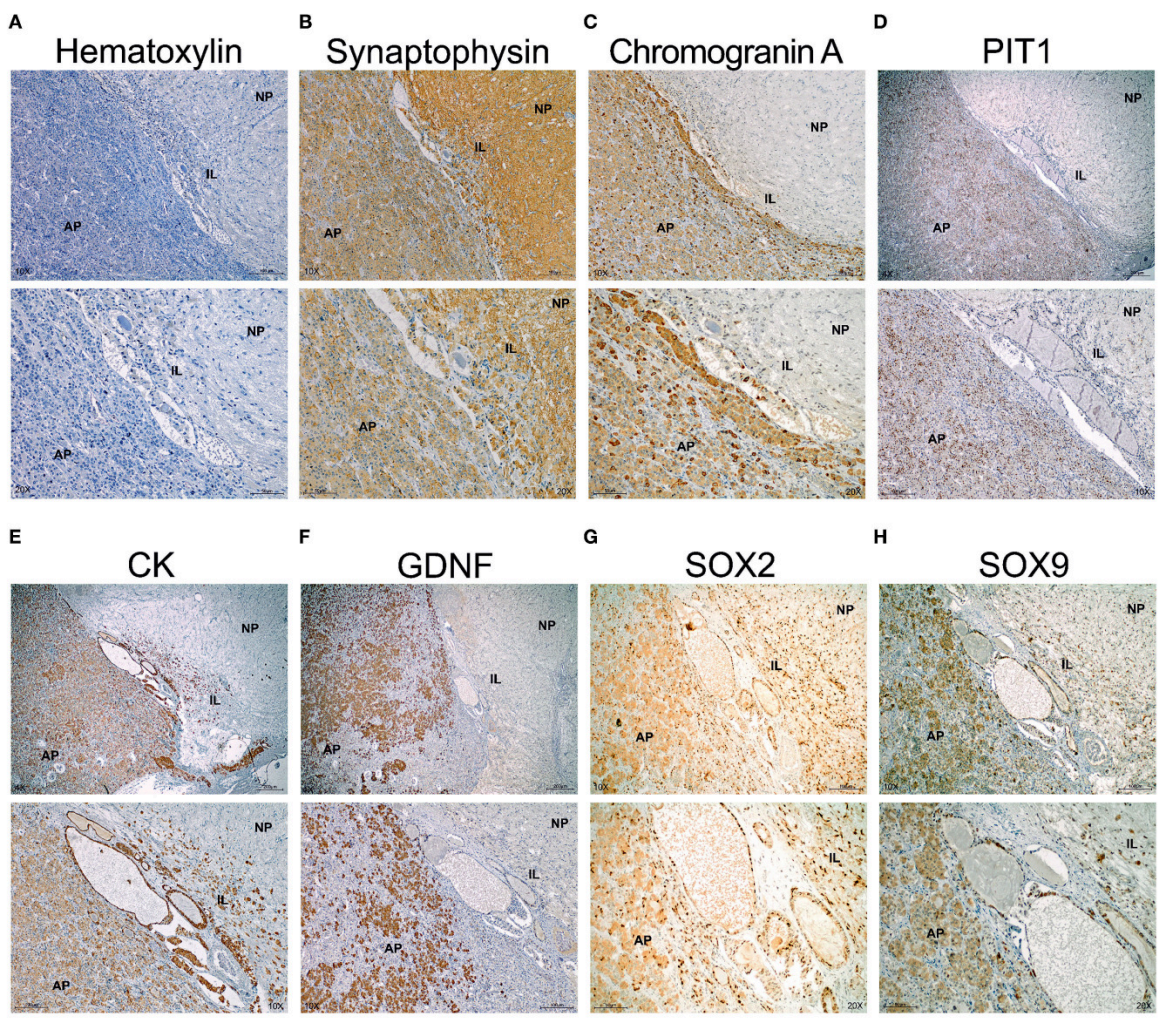
The normal human pituitary co-express GPS stem cell markers at the epithelium of the RC. Immunohistochemistry in sagittal sections of human pituitary counterstained with Haematoxylin to reveal nuclei. Two magnifications are shown per field. **(A)** Negative control omitting primary antibody. No signal is seen and only remains the haematoxylin counterstain revealing the three portions of the pituitary. At the border between the IL and AP appears cysts (Rathke's Cysts, RC), surrounded by a single layer of cells considered the GPS in humans. **(B)** Synaptophysin stains the NP, IL and less the AP. **(C)** Chromogranin A stains the AP. **(D)** PIT1 is a transcription factor express by differentiated somatotrophs, lactotrophs and thyrotrophs at the AP. **(E)** Cytokeratins (CK) are expressed by the epitheial endocrine cells. However, at the niche, the pituitary stem cells overexpress cytokeratins. **(F)** GDNF is the most abundant ligand of RET expressed at the adenopituitary. **(G)** SOX2 is a transcription factor expressed in many cells throughout the NP, IL, and AP. However, intensity is high at the stem cell niche formed by RC. **(H)** SOX9 is a transcription factor expressed by some cells at the NP and AP. Intensity is higher at the RC epithelium. AP, Adenopituitary; IL, Intermediate Lobe; NP, Neuropituitary.

The human pituitary has a small IL comprised between the NP and the AP, the main endocrine portion (Figure 5A). The stem cell niche is located between the IL and the AP at the center of the pituitary and is only visible using sagittal sections through a central longitudinal plane from top to bottom. In the niche, cysts known as Rathke's Cysts (RC) were surrounded by the stem cells (Figure 5A). Immunohistochemistry (IHC) for Synaptophysin delineated the NP and the IL, with less intensity at the AP (Figure 5B). The RC appeared weakly stained. Chromogranin A stained exclusively the AP (Figure 5C). PIT1 the transcription factor common to somatotrophs, lactotrophs, and thyrotrophs stained cells within the AP but none in the marginal zone where the RC were located, nor in the IL or NP (Figure 5D). Cytokeratins are expressed by the all endocrine epithelial cells but its expression was very high in the stem cells that surround the RC in the marginal zone (Figure 5E). GDNF the most abundant RET ligand in the pituitary was expressed exclusively at the AP, with no staining in the IL or NP (Figure 5F). It is known that GDNF is expressed by normal somatotrophs together with GFRα1 and its expression is preserved in somatotroph adenomas (18, 19, 22). SOX2 was intensely detected within the nuclei of the RC epithelium, as corresponds to a marker of GPS stem cells (Figure 5G). However, SOX2 was also expressed by some cells of the NP and IL, and some cells in the AP (Figure 5G). It has been previously shown that recruited SOX2 positive cells migrate from the niche into the AP (27, 28). Similar staining was found for SOX9 (Figure 5H), although its intensity was higher in the RC cells than the AP, IL, or NP.

**Figure 6:**
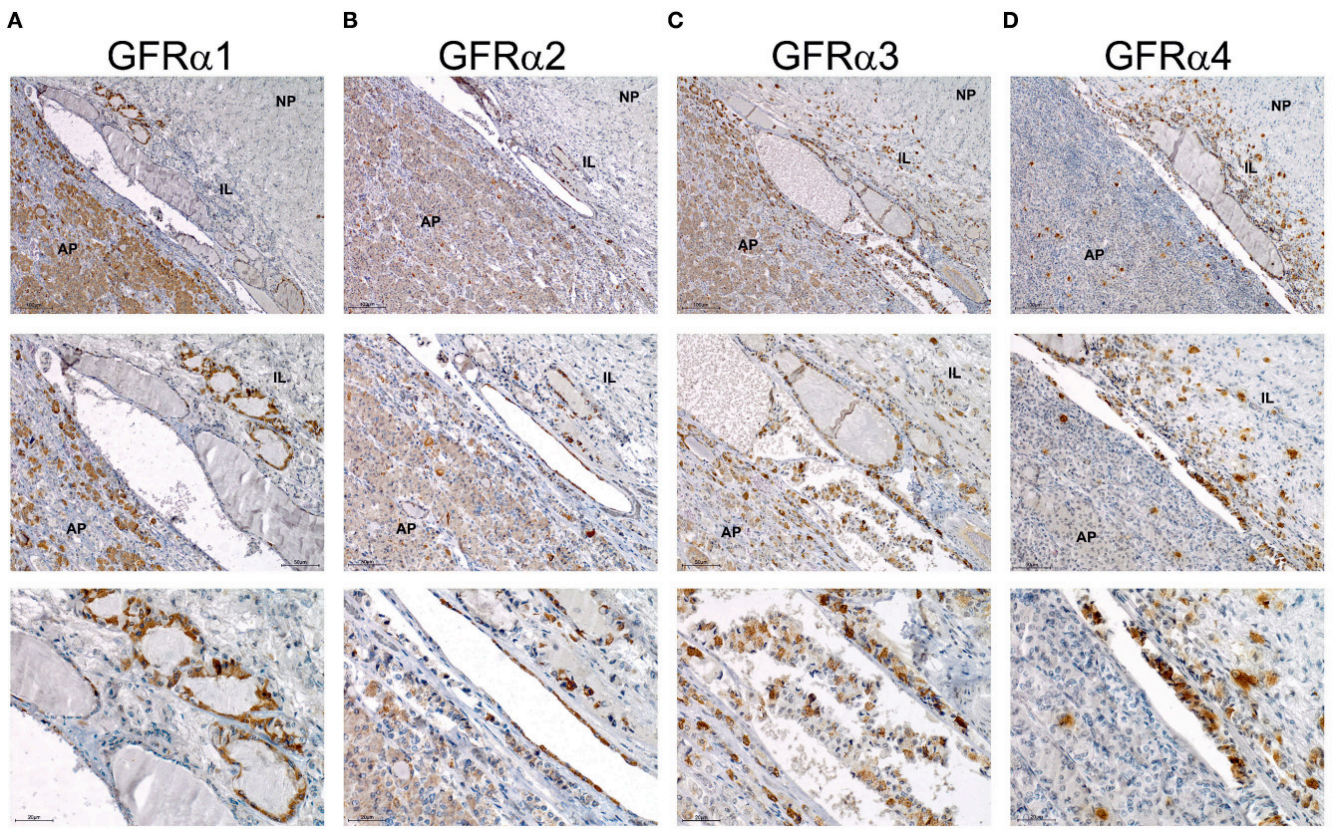
Expression of the GFRα 1-2-3 and 4 co-receptors in human pituitary. Immunohistochemistry of each co-receptor in sagittal sections of human pituitary counterstained with Haematoxylin to reveal nuclei. Three magnifications are shown per field. Top row pictures show a global vision of pituitary and general distribution of the four GFRα co-receptors. Middle row has a higher magnification in order to focus specially in intermediate lobe and adenopituitary. Bottom row shows a close-up view of the stem cell niche composed of RC. **(A)** GFRα1 is abundantly expressed at the AP but intensity at the niche is higher. At highest magnification intensely stained RCs are shown. **(B)** GFRα2 is highly expressed at the niche with some isolated cells through the AP. At highest magnification intensely stained RCs are shown. **(C)** GFRα3 is highly expressed at the niche. Few isolated cells are present at the AP but also at the IL. At highest magnification intensely stained RCs are shown. **(D)** GFRα4 presents a similar staining with high intensity at the niche, and isolated cells in the AP and IL. At highest magnification intensely stained RCs are shown. AP, Adenopituitary; IL, Intermediate Lobe; NP, Neuropituitary.

Next, we performed staining for the RET co-receptors in this human pituitary. IHC for GFRα1 showed an abundant signal in the AP but also specific and intense staining in the epithelium forming RC, which are considered the GPS stem cells (Figure 6A). GFRα2, GFRα3, and GFRα4 were detected at high intensity in the epithelium of the RC (Figures 6B–D). Furthermore, the three co-receptors presented isolated cells within the AP positive for one of these co-receptors (Figures 6B–D). GFRα3 and GFRα4 –but not GFRα1 or GFRα2 also showed individual cells stained within the IL (Figures 6C,D).

## Discussion

In this study we have found that the four RET co-receptors GFRα1-2-3 and 4 are co-expressed in the pituitary stem cells of the rat and human pituitary. These results suggest that the co-expression of the complete RET/GFRα system is important to maintain the stemness condition and that when migrating from the niche toward differentiation, the expression of the co-receptors is progressively lost. Moreover, in both human and rat pituitaries, few isolated cells positive for one or the other receptor could be observed, especially in the human pituitary. There are SOX2 cells in the adenopituitary that are negative for stem cell markers, acting as transit amplifying cells (15, 26, 34, 40, 41). The cells scattered throughout the AP expressing one of the four GFRα co-receptors could be transit amplifying cells, cells committed to differentiation but not yet fully differentiated, or differentiated cells. The last two possibilities could be applied to GFRα1+ cells within the AP since they express PIT1 and GH (18), but not nuclear SOX9. However, GFRα3 or GFRα4 individual cells are negative or present cytoplasmic SOX2, therefore the possibilities are still open.

The importance of GFRα1 in the AP was previously described (1, 20). GFRα1 plays a key role as a co-receptor for GDNF by mediating the action of this ligand on the survival and apoptosis of somatotroph cells through the RET/GDNF-AKT survival pathway (1, 20). However, we have demonstrated expression of GFRα1 in stem cells from the pituitary niche in human and rat, co-expressing RET and the GPS marker β-catenin (15, 39). β-catenin is a well-known marker of epithelial stem cell niches in many organs (38). Our data reveal the expression and colocalization of RET and the four GFRα co-receptors with β-catenin in the stem cell niche of human and rat pituitary. We know that RET and GFRα3 expression is lost in craniopharyngiomas (39), a pituitary benign neoplasia possibly derived of some stem cell population where β-catenin activation and nuclear localization (28), either through mutation (42) or BRAF kinase activation (43), has been described. There is no description of a direct interaction between RET and β-catenin in pituitary cells. However, molecular studies in human HEK293 and neuroblastoma cell lines demonstrated a GDNF/GFRα1-induced interaction between RET and β-catenin at the plasma membrane, resulting in tyrosine phosphorylation of β-catenin which prevents its proteasomal degradation, and results in cell survival and proliferation (44). Future studies will have to explore the RET-β-catenin interaction in pituitary GPS cells.

Stem cell niches are structures where postnatal stem cell reside surrounded by other population that help to maintain their integrity and its proper activation and turnover. The expression of GFRα1 in pituitary stem cells and also somatotrophs (18, and this manuscript) suggest that GFRα1 is conserved during somatotroph differentiation but lost in the rest of hormone-producing cells. Moreover, GFRα1 expression together with its ligand GDNF-remains relevant in somatotroph derived tumors causing acromegaly (18, 19, 45). The increased expression of GDNF, blocks the RET /PIT1/p14ARF/p53 apoptosis pathway inducing AKT activation and survival contributing to cell growth and tumor development (19, 21, 22). Pituitary stem cells are not the unique expressing GFRα1, since is expressed in Sertoli and Leydig cells in testis (Leydig cells also co-express GFRα2), where it arises as essential in spermatogonial stem cells (46). In the eye, the basal layer from the bulbar conjunctiva epithelium express several stem cell markers, including GFRα1, postulating them as a stem cell niche in the conjunctive (47).

GFRα2 in highly and continuously expressed by the GPS (GFRα2, PROP1, Stem) cells at the pituitary niche (13, 15, 39). However, the AP is less enriched in this co-receptor where many of the scattered GFRα2 positive cells belong to follicles that are niche extensions into the AP, and co-express β-catenin (13). Cells from follicles also co-express the other GPS stem cell markers and S-100. Differentiated secretory cells in the adenopituitary are negative for GFRα2, but they are derived from the stem cell niche (27, 28). The mechanism involved in differentiation from stem cell to hormone-producer cell remains unknown. GFRα2 is also transiently expressed in cardiac progenitors which are pluripotent stem cells. Interestingly, GFRα2 knockout ESC cell lines complete cardiomyocyte differentiation. Ishida and collaborators described the redundant role of the GFRα co-receptors, since GFRα1 together with neurturin are able to substitute the lack of GFRα2 in this cardiac progenitors (48). The same could happen in the pituitary and the redundancy of GFRα co-expression is a self-supportive mechanism which ensures proper differentiation.

GFRα3 and GFRα4 were discovered later than GFRα1-2 and thus, they are less studied. GFRα3 and GFRα4 expression patterns found in the pituitary were similar, especially in human pituitary, where scattered cells expressing any of the two co-receptors were not only found in AP but also in IL. It has been described that human pituitary cells do not express GFRα4 (30). However, we demonstrate here that GFRα4 is expressed at both the RNA and protein levels in human and rat pituitary. Moreover, GFRα4 is co-expressed with the other co-receptors in stem cells and is retained in apparent progenitors both at IL and at the AP. All co-receptors are co-expressed together with RET at the niche. Our results with some nuclear markers of commitment or differentiation like PIT1 indicate that cells expressing one single co-receptor in the AP could be marked for commitment to differentiation.

Further studies in individual GFRα co-expression with markers of differentiation would help to delineate the process of recruitment and differentiation leading to a wider comprehension of cell lineage differentiation within the pituitary and to a deeper knowledge of the differentiation process. This has implications in pituitary pathology as demonstrated for the role of RET and GFRα4 in acromegaly tumors (19).

## Data Availability Statement

All datasets presented in this study are included in the article/Supplementary Material.

## Ethics Statement

The studies involving human participants were reviewed and approved by Biobanco, Complexo Hospitalario Universitario de Santiago de Compostela (CHUS). The patients/participants provided their written informed consent to participate in this study. The animal study was reviewed and approved by Servicio de Ganderia, Delegación Territorial da Coruña, Xefatura Territorial da Conselleria do Medio Rural e do Mar, Xunta de Galicia.

## Author Contributions

AP performed animal experiments and stainings. MC performed human staining. MG-L performed stainings. SP-R and MS-F performed the RNA experiments. AG-R performed animal experiments. JC-T collected human samples. IB and CA conceptual design and funding. AP, MC, IB, and CA manuscript and figure preparation. AP, MC, SP-R, AG-R, MS-F, MG-L, JC, IB, and CA final corrections and revision of Ms. All authors contributed to the article and approved the submitted version.

## Conflict of Interest

The authors declare that the research was conducted in the absence of any commercial or financial relationships that could be construed as a potential conflict of interest.
