# Supplementary material for: GFRα 1-2-3-4 co-receptors for RET Are co-expressed in Pituitary Stem Cells but Individually Retained in Some Adenopituitary Cells
Source: Front Endocrinol (Lausanne). 2020 Sep 24;11:631. doi: 10.3389/fendo.2020.00631 (PMC7543094; doi:10.3389/fendo.2020.00631)
Supplement: Supplementary file 1 [file Table_1.DOCX]

**Supplementary table 1.** Primers used for qRT-PCR assay in human and rat.

| Gene | Human (H)  Rat (R) | Sequence | Amplification size | Annealing Tª |
| --- | --- | --- | --- | --- |
| TBP | H | Fw: 5’- GCCCGAAACGCCGAATAT -3’ | 67 bp | 60ºC |
|  |  | Rv: 5’- TTCGTGGCTCTCTTATCCTCATG -3’ |  |  |
|  |  | Pb: 5’- TCCCAAGCGGTTTGCTGCGGTA -3’ |  |  |
| GFRa1 | H | Applied Biosystems: Hs00237133_m1 | 69 bp | 60ºC |
| GFRa2 | H | Applied Biosystems: Hs00176393_m1 | 88 bp | 60ºC |
| GFRa3 | H | Applied Biosystems: Hs00181751_m1 | 89 bp | 60ºC |
| GFRa4 | H | Applied Biosystems: Hs00360831_g1 | 152 bp | 60ºC |
| RET | H | Applied Biosystems: Hs04259657_s1 | 110 bp | 60ºC |
| GH | H | Applied Biosystems: Hs00236859_m1 | 82 bp | 60ºC |
| PRL | H | Applied Biosystems: Hs00168730_m1 | 76 bp | 60ºC |
| POMC | H | Applied Biosystems: Hs01596743_m1 | 142 bp | 60ºC |
| FSHβ | H | Fw: 5'-TTGGTGTGCTGGCTACTGCT-3’ | 115 bp | 60ºC |
|  |  | Rw: 5'-GGGCACTCTCACTGTTTCGT-3’ |  |  |
| TSHβ | H | Fw: 5'-ATTGCCTAACCATCAACACCAC-3’ | 102 bp | 60ºC |
|  |  | Fw: 5'-AAACATCCTGGGACAGAGCATA-3’ |  |  |
| GHRHR | H | Applied Biosystems: Hs00173457_m1 | 104 bp | 60ºC |
| SSTR2 | H | Fw: 5'-GGCATGTTTGACTTTGTGGTG-3' | 185 bp | 60ºCb |
|  |  | Rw: 5'-GTCTCATTCAGCCGGGATTT-3' |  |  |
| SSTR5 | H | Fw: 5'-CTGGTGTTTGCGGGATGTT-3' | 183 bp | 60ºC |
|  |  | Rw: 5'- GAAGCTCTGGCGGAAGTTGT-3' |  |  |
| Tbp | R | Fw: 5’-CTTCGTGCCAGAAATGCTGAA-3’ | 79 bp | 60ºC |
|  |  | Rv: 5’-CAGTTGTTCGTGGCTCTCTTATTCTC-3’ |  |  |
|  |  | Pb: 5’-AATCCCAAGCGGTTTGCTGCAGTCA-3’ |  |  |
| Gfra1 | R | Fw: 5’-GGCGGCACCATGTTCCTA-3’ | 116 bp | 60ºC |
|  |  | Rv: 5’-CACTGATCACTGGCTTTCACACA-3’ |  |  |
|  |  | Pb: 5’-CCTGGATTTGCTGATGTCGGCCG-3’ |  |  |
| Gfra2 | R | Fw: 5’-TGTCATCACCACCTGCACATCT-3’ | 101bp | 60ºC |
|  |  | Rv: GACTGATGTTTGTCGTGAGCTCTGT-3’ |  |  |
|  |  | Pb: 5’-AGGAGCAAGGGCTGAAGGCCAACA-3’ |  |  |
| Gfra3 | R | Fw: 5’-GCCGCTAGTGATCCTGCTACTG-3’ | 77 bp | 60ºC |
|  |  | Rv: TGTTCTCTGTGGGAAGGGAGTTT-3’ |  |  |
|  |  | Pb: 5’-TCGCTGTGGCTACCCCTTGGAACA-3’ |  |  |
| Gfra4 | R | Fw: 5’-TACGGGTGCTGAATGAGAGG-3’ | 276 bp | 60ºC |
|  |  | Rv: AGAGCAGCGCGTGCGTGAG-3’ |  |  |
