# Supplementary material for: GFRα 1-2-3-4 co-receptors for RET Are co-expressed in Pituitary Stem Cells but Individually Retained in Some Adenopituitary Cells
Source: Front Endocrinol (Lausanne). 2020 Sep 24;11:631. doi: 10.3389/fendo.2020.00631 (PMC7543094; doi:10.3389/fendo.2020.00631)
Supplement: Supplementary file 2 [file Table_2.DOCX]

**Supplementary table 2.** Antibodies used in IHC and IF staining.

| **1^st^ Antibody** | **Company** | **Origin** | **Dilution / Incubation** | | | |
| --- | --- | --- | --- | --- | --- | --- |
|  |  |  | **IHC** | **IF** | | |
| **β-catenin (Clone β-Catenin-1)** | DAKO, M3539 | Mouse | - | 1:500**  ON 4ºC | | |
| **RET_L_ (C-20)** | Santa Cruz, sc-1290 | Goat | - | 1:100  3%BSA in PBS  ON 4ºC | | |
| **RET** | Sigma, HPA008356 | Rabbit | - | 1:500 PBS  ON 4ºC | | |
| **GFRα1 (C-20)** | Sta.Cruz Biotech, sc-6157 | Goat | 1:200* / 12 h 4ºC | 1:50  3%BSA in PBS  ON 4ºC | | |
| **GFRα2** | Chemicon, AB5141 | Rabbit | 1:200* / 12 h 4ºC | 1:1000 PBS  ON 4ºC | | |
| **GFRα3 (V-19)** | Sta. Cruz Biotech, sc-9340 | Goat | 1:200* / 12 h 4ºC | 1:100  3%BSA in PBS  ON 4ºC | | |
| **GFRα4** | Signalway Antibody, #31272 | Rabbit | 1:400* / 12 h 4ºC | 1:50 PBS  ON 4ºC | | |
| **PIT1 (D-7)** | Sta. Cruz Biotech, sc-393943 | Mouse | - | 1:100 PBS  ON 4ºC | | |
| **SOX2** | Invitrogen, MA1-014 | Mouse | - | 1:1000 PBS  ON 4ºC | | |
| **SOX2** | Seven Hills, WRAB12136 | Rabbit | 1:1000* / 12 hours (h) 4ºC | - | | |
| **SOX9** | Sigma, HPA001758 | Rabbit | - | 1:1000 PBS  ON 4ºC | | |
| **SOX9 (H-90)** | Sta. Cruz Biotech, sc-20095 | Rabbit | 1:500* / 12 h 4ºC | - | | |
| **GDNF (D-20)** | Sta. Cruz Biotech, sc-328 | Rabbit | 1:500* / 12 h 4ºC | - | | |
| **PIT1 (X-7)** | Sta. Cruz Biotech, sc-442 | Rabbit | 1:500* / 12 h 4ºC | - | | |
| **CK (Clone AE1/AE3)** | DAKO, IR053 | Mouse | Ready to use  / 1h 25ºC | - | | |
| **Chromogranin A** | DAKO, IR502 | Rabbit | Ready to use  / 1h 25ºC | - | | |
| **Synaptophysin**  **(Clone DAK-SYNAP)** | DAKO, IR660 | Mouse | Ready to use  / 1h 25ºC | - | | |
| **2^nd^ Antibody** | **Company** | **Origin** | **Dilution / Incubation** | | | |
|  |  |  | **IHC** | | **IF** |  |
| **Anti-Rabbit-Cy3** | Jackson IR, 711-166-152 | Donkey | - | 1:1000 | | |
| **Anti-Mouse-A488** | Jackson IR, 715-546-151 | Donkey | - | 1:1000 | | |
| **Anti-Mouse-Cy3** | Jackson IR 715-166-151 | Donkey | - | 1:1000 | | |
| **Anti-Mouse-A647** | Jackson IR 715-606-151 | Donkey | - | 1:1000 | | |
| **Anti-Goat-A488** | Jackson IR, 705-546-147 | Donkey | - | 1:1000 | | |
| **EnVision^TM^ FLEX/HRP system** | DAKO, K4061 | - | Ready to use / 30 min 25ºC | - | | |
| **Antibodies were diluted in EnVision FLEX Antibody diluent (DAKO, DM830)*  ***b-catenin was incubated in PBS when prepared together with GFRa2 and GFRa4 antibodies. Together with GFRa1, GFRa3 and RET(C-20), it was incubated in PBS with 3% BSA.* | | | | | | |
